# Enhancing *Eucalyptus* seedling quality through multi-strain bacterial inoculation

**DOI:** 10.1007/s11274-026-04960-8

**Published:** 2026-04-29

**Authors:** Millena Salles Araujo, Igor Juliano da Silva Souza, Ricardo Previdente Martins, Carlos Carneiro dos Santos, Reginaldo Gonçalves Mafia, Caio Tavora Coelho da Costa Rachid

**Affiliations:** 1 LABEM - Laboratory of Biotechnology and Microbial Ecology, Universidade Federal do Rio de Janeiro, Rio de Janeiro, Brazil; 2 Faculdade de Ciências Agronômicas, Universidade Estadual Paulista, Botucatu, SP 18610-034 Brazil; 3 Bracell Florestal, Pesquisa e Desenvolvimento Florestal, Rua Dr. José Tiago Correia, Alagoinhas Velha, Alagoinhas, BA 48030-480 Brasil

**Keywords:** Microbial consortium, Plant growth-promoting bacteria (PGPB), Root community composition, Forest sustainability, Inoculant effectiveness

## Abstract

The use of bacterial inoculants in forestry is a promising strategy to enhance seedling quality, reduce costs, and promote sustainable cultivation. This study evaluates the effects of a multi-strain bacterial consortium on *Eucalyptus* seedlings, considering the plant growth, microbial community composition, and seedling quality. A consortium consisting of one representative from each of five bacterial genera - *Bacillus*,* Paenibacillus*,* Paraburkholderia*,* Methylobacterium*, and *Mesorhizobium* - was applied to three *Eucalyptus* clones, a large field experiment was conducted in a tree nursery. To assess the root endophytic community composition, samples were processed and analyzed using 16 S rRNA metabarcoding sequencing to characterize the bacterial communities present. After 13 weeks, we assessed microbial diversity, morphological seedling traits, and survival rates. While inoculation did not significantly affect bacterial richness and diversity, it altered the root microbial community structure. Two genotypes exhibited improved seedling quality, with a higher proportion classified into superior categories, and one genotype showed a significant reduction in mortality (from 21.8% to 6.7%). Additionally, inoculated seedlings displayed reduced relative abundance of *Ralstonia*, a genus that includes vascular phytopathogenic species responsible for *Eucalyptus* wilt disease. Biomass allocation patterns varied, indicating genotype-specific responses to inoculation. These findings underscore the potential of bacterial consortia to enhance *Eucalyptus* seedling resilience and productivity, presenting a sustainable alternative to conventional forestry practices.

**Supplementary Information:**

The online version contains supplementary material available at 10.1007/s11274-026-04960-8.

## Introduction

*Eucalyptus* is the predominant genus in Brazilian silviculture, occupying 74% (7.8 million hectares out of 10.5 million hectares) of the cultivated area in 2023 (IBÁ 2024). Plantations are distributed throughout the Brazilian territory, especially in the Southeast and Midwest regions of the country. The use of the *Eucalyptus* genus as a source of raw material is due to its attributes, such as low nutritional demand, rapid growth compared to other crops, high planting density, trunk shape, quality of obtained wood, ability to regrow, and easy seedling production (Embrapa 2019; Surbhi et al. 2023), as well as good adaptation to the country’s soil and climate conditions.

Planted forest areas in Brazil represent less than 1% of the total territory, with *Eucalyptus* occupying most of this cultivated area. However, these planted forests supply 91% of all wood used for productive purposes, especially cellulose production (Embrapa 2019). This remarkable productive potential is reflected in the performance indicators of national exports, consolidating Brazil as the world’s largest exporter of pulp in 2021 (IBÁ 2023). Although the planted area sector reached record production levels, it faced significant increases in the cost of raw material production. From December 2020 to December 2021, wood production costs increased by 94%, which created a major obstacle to maintaining profitability in the forestry industry (IBÁ 2022). Factors such as pest and disease infestations, adverse weather conditions, and lack of proper management can also have a negative impact on forest production levels (Booth 2013; Guo et al. 2023).

Given its economic importance and the constant need for cellulose-derived products, new technologies are sought to maintain and/or increase productivity while reducing the use of chemical inputs. This results in more sustainable cultivation and higher profits (Eggers et al. 2020).

Several studies demonstrate a positive impact on plant growth and development, as well as increased tolerance to potential environmental stress, using plant growth-promoting bacteria (Backer et al. 2018; Nwigwe et al. 2023). These microorganisms have been shown to contribute to phytohormone production, nutrient solubilization and mineralization, iron chelation through siderophore production, and the synthesis of metabolites that inhibit the growth of phytopathogens and plant defense responses (Bashan and De-Bashan 2005; Cardoso and Andreote 2016; Olanrewaju et al. 2017; Gouda et al. 2018; Chaín et al. 2020). Microorganisms that colonize the internal tissues of plants, known as endophytes, establish a more intimate and stable systemic interaction with the host. This close association favors the modulation of physiological and metabolic processes, often resulting in significant effects on plant nutrition, root system architecture, and tolerance to both biotic and abiotic stresses (Abbamondi et al. 2016; Afzal et al. 2019). Recognizing the beneficial potential of microorganisms when applied to plants, the use of these microorganisms in the form of inoculants is proposed for more sustainable plantations (Gouda et al. 2018).

The application of microbial inoculants is a well-established practice in several crops, including soybean, corn, and sugarcane. For instance, inoculation of soybeans with *Bradyrhizobium japonicum* has consistently enhanced nodulation and productivity (Henrique et al. 2019). In sugarcane, inoculation with *Bacillus subtilis* and *Pseudomonas fluorescens* has improved phosphorus acquisition, allowing a reduction of up to 75% in the recommended P₂O₅ fertilization rate (Rosa et al. 2022). For use in forestry plantation areas, several studies have been carried out. For example, strains of rhizobacteria isolated from *Eucalyptus* spp. have been shown to promote rooting through an increase in root biomass and growth of *Eucalyptus* cuttings (Mafia et al. 2007a, b) and the reduction of *Cylindrocladium* cutting rot (Mafia et al. 2009), rust infection caused by *Puccinia psidii* (Teixeira et al. 2005) and bacterial wilt caused by *Ralstonia solanacearum* (Santiago et al. 2015).

Evidence has indicated that inoculating *Pinus taeda* seedlings with growth-promoting bacteria, such as *Bacillus subtilis*, significantly increases root and shoot biomass, thereby enhancing adaptation to field conditions (Santos et al. 2018). Furthermore, bacterial inoculation was shown to promote rooting in difficult-to-root *Eucalyptus* cuttings, achieving rates comparable to treatments with indole-3-butyric acid (IBÁ) (Nwigwe et al. 2023). Similarly, inoculation with nitrogen-fixing bacterial strains resulted in increased plant height and diameter (Souza et al. 2022). Taken together, these findings suggest that adopting inoculants in forestry could provide environmental, social, and economic advantages, including increased productivity, reduced costs, lower dependence on imported inputs, and improvements in soil health and quality (Sammauria et al. 2020; Gomes et al. 2025).

To complement these studies, it is necessary to evaluate the bacterial community in seedlings to understand how inoculants reshape root-associated microbial communities over time. This knowledge gap is particularly critical in forestry, where in a large field experiment conducted in a tree nursery and genotype-specific responses demand integrative approaches that couple plant performance with the dynamics of bacterial community composition. Addressing these limitations is essential to evaluate not only the direct growth-promoting effects of inoculants but also their long-term ecological compatibility and potential to enhance resilience in *Eucalyptus* cultivation.

In previous studies (Fonseca 2014, 2020), a bacterial consortium composed of five strains from the genera *Bacillus*,* Paenibacillus*,* Paraburkholderia*,* Methylobacterium*, and *Mesorhizobium* was developed. These bacteria were selected for their strong performance in phosphate solubilization tests, and some exhibited the presence of the *nif*H gene (indicating potential nitrogen-fixing capacity) as well as the production of indolic compounds. Under greenhouse conditions, they demonstrated promising results, enhancing plant survival, biomass, and growth.

In this study, we aim to understand the effects of applying the bacterial consortium to seedlings of different *Eucalyptus* genotypes on a large field experiment conducted in a tree nursery. Additionally, we hypothesize that the application of the consortium: (i) would alter the microbial pattern found in the roots of inoculated seedlings compared to non-inoculated seedlings, (ii) would alter the distribution of genera composing the bacterial consortium in the roots compared to non-inoculated seedlings, and (iii) would improve seedling development. These hypotheses are based on the premise that the introduction of beneficial microbial consortia can modulate the native root microbiome, influence the establishment and relative abundance of inoculated taxa, and consequently improve plant growth and development through multiple plant growth-promoting mechanisms.

## Methodology

### Experimental design

Three clonal genotypes of *Eucalyptus* (hereafter referred to as X, Y, and Z) were selected for a large field experiment conducted in a tree nursery to evaluate the effects of bacterial inoculation on seedling performance. The genotypes were selected based on characteristics provided by the partnering company, which reported the following traits: genotype X (*E. urophylla × E. grandis hybrid*) exhibited poor rooting; genotype Y (*E. urophylla*) presented rapid post-planting growth; and genotype Z (*E. urophylla*) showed a high incidence of apical shoot abortion. These contrasting phenotypic properties provided a suitable basis for assessing plant–microbe interactions across variable host responses.

The experiment followed a factorial design with two factors: genotype (X, Y, and Z) and inoculation (with and without the bacterial inoculant), totaling six treatments. The six treatments were arranged in six randomized blocks; within each block, each treatment comprised 176 seedlings (1,056 seedlings per treatment). For each genotype, 2,112 seedlings were assessed, 1,056 inoculated and 1,056 uninoculated, resulting in a total of 6,336 seedlings in the experiment.

Control seedlings received the same management conditions as inoculated plants, except that irrigation was performed using water without bacterial inoculum.

## Inoculant preparation

To prepare the inoculum, five bacterial strains belonging to the genera *Bacillus*, *Paenibacillus*, *Paraburkholderia*, *Methylobacterium*, and *Mesorhizobium* were individually cultured in a liquid medium (Phytate Low), following protocols described by Fonseca (2018, 2020) and Salles (2021). Cultures were incubated at 30 °C for 48 h under constant agitation at 150 rpm. The bacterial strains were originally isolated from the rhizosphere and roots of *Eucalyptus* spp. and had their taxonomy determined and characterized in previous studies (Fonseca 2014, 2020; Fonseca et al. 2018).

The consortium comprises five strains, including *Bacillus subtilis*, *Paraburkholderia caribensis*, and *Methylobacterium tardum*, as well as two strains representing putative novel species within the genera *Paenibacillus* and *Mesorhizobium*, based on metagenome-assembled genome (MAG) analyses. The strains were selected to form a synthetic community based on their complementary functional traits related to nitrogen cycling and rhizosphere colonization. In previous studies, these strains were primarily evaluated as a consortium rather than as individual inoculants (Fonseca 2014, 2020; Fonseca et al. 2018).

After incubation, bacterial growth was quantified by measuring optical density (OD) at 600 nm using a spectrophotometer. Based on OD values, the required volume of each culture was harvested and centrifuged at 10,000 × g for five minutes to concentrate the cells and placed into duplicate microtubes and then transported separately to the experimental site. Based on OD measurements, the required volume of each culture was harvested and centrifuged at 10,000 × g for 5 min to concentrate the cells. The resulting pellets were resuspended in phosphate-buffered saline (PBS 1×), and all strains were adjusted to the same optical density. Equal volumes of each standardized suspension were then combined to prepare the consortium in a final volume of ten L of PBS (137 mM NaCl, 2.7 mM KCl, 10 mM Na₂HPO₄, 1.8 mM KH₂PO₄; pH 7.4). Following mixing and dilution, the final solution reached an OD of 0.1 (approximately 10⁷ CFU mL⁻¹ per strain), as described by Fonseca (2020).

## Experimental setup

The experiment was conducted at a forestry company facility located in Entre Rios, Bahia, Brazil. Vegetative propagation material for the genotypes consisted of mini-cuttings derived from mini-stumps maintained in a clonal mini-garden under a semi-hydroponic system utilizing sand channels. Immediately after collection, mini cuttings were stored in thermal boxes to maintain viability until planting.

Propagation was carried out in 55 cm³ polypropylene tubes filled with Carolina Soil^®^ commercial substrate, which were then irrigated. The bacterial inoculant, pre-diluted in PBS, was applied to the substrate in each tube using a watering can ensuring uniform distribution. The tubes were subsequently arranged in trays and maintained under nursery conditions for seedling development.

Seedlings were cultivated under standard nursery conditions, progressing through distinct developmental phases. During the clonal mini-garden phase, plants were maintained under ambient conditions, with relative humidity typically above 80% and an average temperature of approximately 23 °C. After planting, seedlings were transferred to greenhouse conditions, where relative humidity was maintained close to saturation (~ 100%) and temperature was controlled between approximately 25 and 32 °C through automated ventilation systems; this phase lasted 20 days. Subsequently, seedlings were moved to a shade house (30% shading) for acclimation, remaining under these conditions for an additional 20 days under ambient temperature and humidity. Finally, seedlings underwent a hardening phase under ambient environmental conditions for 50 days, completing a total nursery period of 91 days.

## Monitoring

The experiment lasted for 13 weeks, during which seedlings were periodically evaluated and classified by size. At the conclusion of the experiment, seedlings were grouped into three categories based on morphological development: Category A - Seedlings exhibiting vigorous growth and optimal structural characteristics; Category B - Seedlings displaying moderate growth, within acceptable standards for field planting; Category C - Seedlings with insufficient development, not meeting the minimum criteria for field deployment.

Seedlings classified as A and B were considered suitable for field transplantation. In contrast, category C seedlings remained in the nursery under extended cultivation to improve growth and potentially achieve reclassification. Seedlings exhibiting severe stem curvature (defined as a deviation greater than 45° from the vertical axis) or found dead during the evaluation period were counted as dead. At the end of the 13-week period, a subset of seedlings was randomly selected from each treatment group for detailed morphological analysis. Specifically, 180 seedlings classified as Category A and 120 seedlings classified as Category B were randomly selected per treatment. An exception was made for genotype X, which exhibited a low number of Category B seedlings; therefore, only 20 seedlings were available and included for further analysis.

To assess plant biomass, the aerial parts of the selected seedlings were separated from the roots and placed in paper bags, then dried in a forced-air oven at 60 °C for 72 h. Root systems were carefully washed with water to remove residual substrate, avoiding damage to fine root structures, and subsequently placed in paper bags for drying under the same conditions. After the drying period, the dry weight of both shoot and root components was determined using an analytical balance.

## Analysis of the endophytic bacterial community in seedling roots

### Root disinfection and processing

To evaluate the effects of the inoculant on endophytic bacterial community of *Eucalyptus* roots seedlings, a subset of seedlings was selected for sequencing metabarcoding of the 16 S rRNA gene. A total of 30 seedlings were randomly selected, comprising three seedlings from Category A and two from Category B per treatment. These samples were collected at the end of the experimental period and sent to the laboratory for molecular analysis.

In the laboratory, root fragments were excised from each seedling using sterile scissors and carefully washed to remove residual substrate and fertilizer adhering to the surface. The samples were then subjected to a disinfection protocol adapted from Araújo et al. (2001), designed to eliminate epiphytic microorganisms while preserving endophytic communities. Initially, roots were rinsed under running water to remove particulate matter and then dried on absorbent paper. This was followed by immersion in 70% ethanol (30 mL) for one minute, then in 2% sodium hypochlorite solution (30 mL) for two minutes. Subsequently, the samples were exposed to ultraviolet (UV) light in a laminar flow hood for five minutes. A second wash in 70% ethanol (30 mL) for one minute was performed, followed by five consecutive rinses in sterile distilled water (30 mL each) to remove any residual disinfectants. After surface sterilization, the root tissues were frozen in liquid nitrogen and macerated using sterile mortar and pestle. The homogenized material was then stored at − 20 °C for subsequent DNA extraction and molecular analyses.

## Extraction and purification of DNA

The homogenized material obtained from root tissue maceration was used for DNA extraction with the PowerSoil^®^ DNA Isolation Kit (MoBio Laboratories, CA, USA), following the manufacturer’s protocol. For each extraction, 200 mg of homogenized root material was used. A negative control, consisting of an extraction without plant tissue, was included to monitor for potential contamination.

Universal bacterial primers 27 F (5′-AGA GTT TGA TCA TGG CTC AG-3′) and 1492R (5′-GTT TAC CTT GTT ACG ACT T-3′) were used to amplify the 16 S rRNA gene (Lane, 1991). Quality control step to verify the efficiency of DNA extraction. PCR products were subsequently subjected to agarose gel electrophoresis (1.6%) to confirm the presence and expected size of the amplified fragments. Following this verification, the PCR products were purified using the Wizard^®^ SV Gel and PCR Clean-Up system (Promega Corporation, USA), according to the manufacturer’s protocol. The PCR products were purified using the Wizard^®^ SV Gel and PCR Clean-Up System (Promega Corporation, USA), following the manufacturer’s instructions. Briefly, the PCR product was mixed with membrane binding solution and transferred to a silica membrane spin column, where the DNA binds to the matrix. The column was centrifuged to allow binding, and the flow-through was discarded. Subsequently, the bound DNA was washed by adding the membrane wash solution, followed by centrifugation to remove contaminants such as primers, nucleotides, enzymes, and salts. This washing step was performed twice to ensure efficient removal of residual impurities. An additional centrifugation step was carried out to remove residual ethanol from the membrane. Finally, the purified DNA was eluted by adding ultrapure water to the column, followed by centrifugation, and the eluate containing high-purity DNA was collected. The concentration and quality of the purified DNA were determined using a Qubit™ 4 fluorometer (Thermo Fisher Scientific^®^), and the DNA was stored at -20 °C until sequencing.

The purified DNA samples were submitted for sequencing of the V5–V7 hypervariable region of the 16S rRNA gene, a region commonly used for profiling bacterial endophytes. Amplification was performed using the primer pair 799F (5’-AACMGGATTAGATACCCKG-3’) and 1193R (5’-ACGTCATCCCCACCTTCC-3’), as described by Redford et al. (2010). PCR conditions were as follows: 98 °C for one min; 30 cycles of 98 °C for ten s, 50 °C for 30 s, 72 °C for 30 s; 72 °C for five min. The 30 µL reactions contained 15 µL of Phusion High-Fidelity PCR Master Mix with GC Buffer (New England Biolabs, USA), three µL of each primer (6 µM), 10 µL of gDNA (5 ~ 10 ng) and two µL of ultrapure H2O. Sequencing was carried out by Novogene Corporation (Sacramento, CA, USA) on the Illumina NovaSeq 6000 platform (100 K paired-end 250 bp reads, Illumina, USA), following the manufacturer’s protocols for library preparation and sequencing.

## Bioinformatics analysis

Metabarcoding DNA sequencing analyses were processed with Mothur v.1.46.1 (Schloss et al. 2009). Primer and barcode sequences were trimmed and paired-end reads were assembled. Low-quality sequences, those containing ambiguous bases, more than eight homopolymers, or lengths outside the expected range (minlength = 372, maxlength = 385), were discarded. High-quality reads were aligned against the SILVA SSU reference database v.138 (Quast et al. 2013), and only those with consistent alignment were retained. All sequences were then trimmed to a common start and end position to ensure comparability. Pre-clustering was applied to merge reads differing by up to three base pairs, followed by chimera detection and removal with chimera.vsearch (Rognes et al. 2016). Taxonomic classification was performed with the Ribosomal Database Project v.18 (Cole et al. 2009) using an 80% bootstrap confidence threshold. Sequences assigned to mitochondria, chloroplasts, Eukarya, Archaea, or unclassified at the Domain level groups were excluded. Remaining reads were clustered into OTUs at a 97% similarity cutoff, and singletons were removed. The OTU distribution matrix was subsequently curated with the decontam R package (Davis et al. 2018) using a combined prevalence- and frequency-based approach to eliminate potential contaminants. Finally, datasets were normalized by rarefaction to the smallest sample size (3,249 reads per sample) to minimize biases arising from uneven sequencing depth.

### Statistical analysis

Alpha-diversity indices (OTU), diversity (Shannon index) underwent Two-Way Analysis of Variance (ANOVA) followed by Tukey’s post-hoc test using Past 5 software (Hammer et al. 2001). Additionally, Levene’s test was conducted to verify homoscedasticity of all data. Mean comparisons between treatments were made considering a probability level of 0.05.

To test differences in taxon abundance at the phylum and genus levels, differential abundance analyses were conducted using ANCOM-BC-2 (Analysis of Composition of Microbiomes with Bias Correction) (Lin and Peddada 2024), which explicitly accounts for the compositional nature of amplicon sequencing data and corrects for sampling fraction bias. All analyses were performed on non-rarefied raw count tables, following current recommendations for microbiome differential abundance testing. Analyses were conducted at both the phylum and genus levels. At the genus level, taxa with extremely low information content were filtered by retaining only genera with a minimum cumulative abundance of ≥ 100 reads across all samples. In addition, taxa showing zero variance within any experimental group (defined by the Inoculation × Genotype combination) were excluded to allow proper model fitting. Differential abundance was assessed using a two-way fixed-effects model with the formula: abundance ~ Inoculation × Genotype, where Inoculation (inoculated vs. control) and Genotype (X, Y, Z) were treated as categorical predictors. P-values were adjusted for multiple testing using the Benjamini–Hochberg false discovery rate (FDR) procedure, and all statistical inferences were based on the resulting adjusted p-values (q-values), using a significance threshold of q < 0.05.

Differential abundance was evaluated using both the standard ANCOM-BC-2 test (diff) and the sensitivity analysis for pseudo-count addition, which assesses the robustness of results to zero handling. Only taxa passing the sensitivity analysis (diff_robust = TRUE) were considered robustly differentially abundant. Taxa identified as significant prior to sensitivity analysis but failing the robustness criterion were classified as non-robust trends and interpreted as exploratory. To further examine genotype effects, additional pairwise genotype contrasts (Y vs. X, Z vs. X, and Z vs. Y) were evaluated using both contrasts derived from the global model and independent two-group ANCOM-BC-2 models adjusted for inoculation.

To illustrate the average relative abundance of community components, a heatmap was created using Past 5 software. In the map, warmer colors (red) represent an increase in a specific taxon, while cooler colors (blue) represent a decrease in that taxon in the treatment studied. The same data were represented using Z-Score, indicating whether a particular number varies relative to the mean of the data. This variation can be below the average (negative Z-score) or above (positive Z-score), visible on the right side of the heatmaps in gray color spheres.

A Non-Metric Multidimensional Scaling (nMDS) analysis was also conducted in two dimensions using Bray-Curtis distance, considering all treatments and by genotypes. Statistical analyses and ordination were performed using Past 4.03 software. A permutational multivariate analysis of variance (PERMANOVA) was carried out, also using the Bray-Curtis dissimilarity index, to test whether the samples differed from each other.

Differences in the distribution of seedling morphological categories (A, B, C, and dead) between inoculated and non-inoculated treatments were evaluated using chi-square tests of independence. Analyses were performed separately for each genotype, as the response variable consisted of categorical frequency data. When significant differences were detected, standardized residuals were examined to identify which morphological categories contributed most strongly to the observed chi-square statistics, allowing a detailed interpretation of treatment effects within each genotype. Statistical significance was assessed at *p* < 0.05.

To analyse the morphological aspects of the plants (height, diameter, number of leaves, biomass weight). Normality of the data was assessed using the Shapiro-Wilk test, which evaluates whether a sample follows a normal distribution. For data that exhibited normal distribution (*p* > 0.05 in the Shapiro-Wilk test), the Student’s t-test was applied. This parametric test is used to compare means between two groups. These data were marked with an asterisk (‘*’). For data that did not follow a normal distribution (*p* < 0.05 in the Shapiro-Wilk test), the Mann-Whitney test was used. This non-parametric test is appropriate for comparing two independent samples when the normality assumption is not met. These data were indicated with a plus sign (‘+’).

Additionally, the graphs present error bars representing the mean ± standard deviation, providing an indication of data variability in each evaluated condition.

The boxplots were generated using the PAST software to visualize data distribution and potential outliers.

## Results

### Shifts in seedling quality classes

**Fig. 1:**
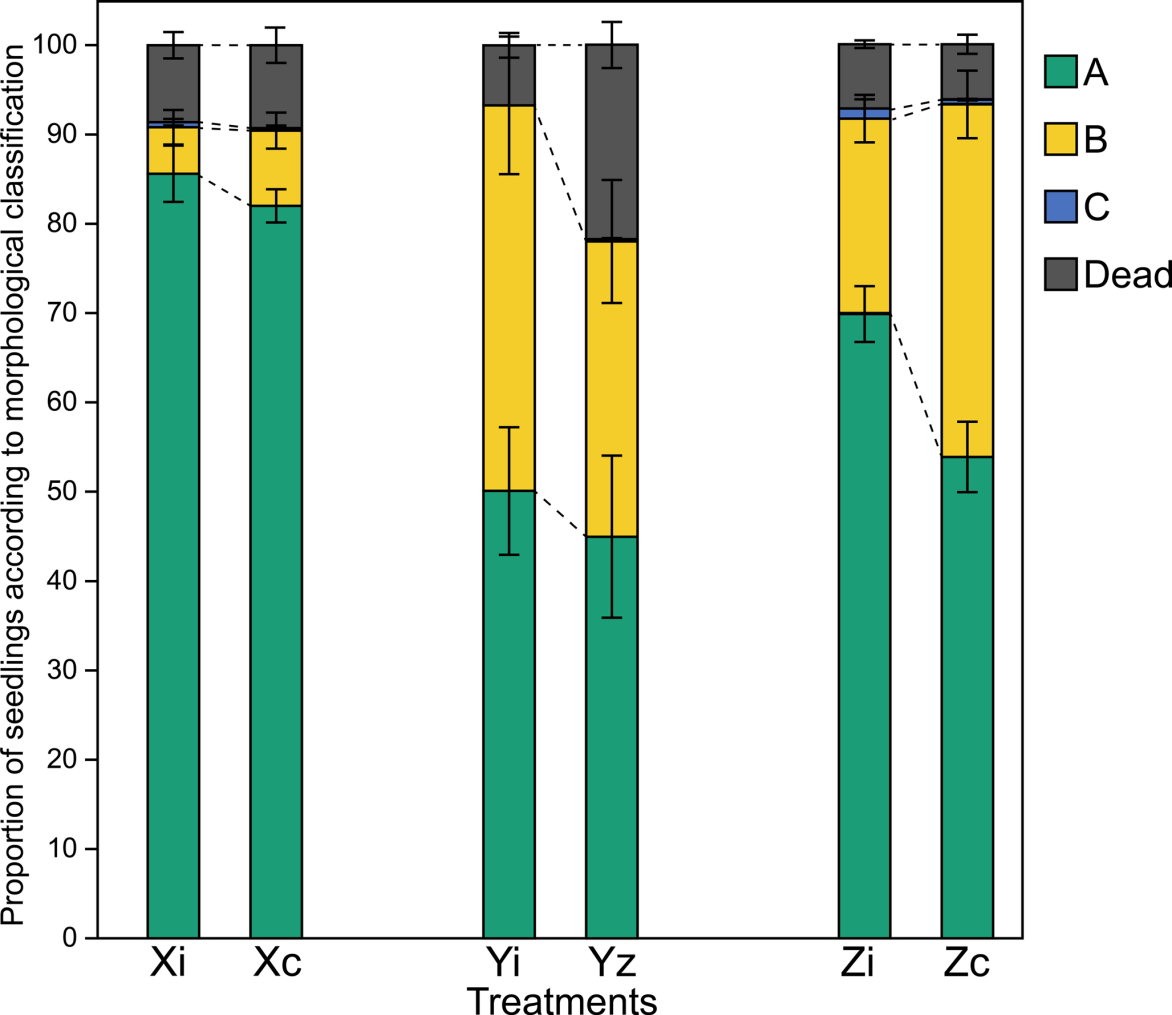
Each bar represents the mean proportion of seedlings in each morphological category, with standard errors, based on six blocks of 176 seedlings per treatment. Treatments correspond to three *Eucalyptus* genotypes (X, Y, and Z), either inoculated (i) or non-inoculated control (c). Category A – seedlings exhibiting vigorous growth and optimal structural traits; Category B – seedlings with moderate growth, within acceptable standards for field planting; Category C – seedlings with insufficient development, not meeting the minimum criteria for field deployment; Dead – non-viable seedlings. Chi-square tests were performed on aggregated observed counts across blocks within each treatment (*n* = 1,056 seedlings per treatment). Asterisks (*) indicate significant differences between treatments (*p* < 0.05). Each bar represents the mean proportion of seedlings in each morphological category, with standard errors, based on six blocks of 176 seedlings per treatment. Category A – seedlings exhibiting vigorous growth and optimal structural traits; Category B – seedlings with moderate growth, within acceptable standards for field planting; Category C – seedlings with insufficient development, not meeting the minimum criteria for field deployment; Dead – non-viable seedlings. Asterisks (*) indicate significant differences between treatments, according to the chi-square test (*p* < 0.05)

The morphological classification of seedlings was assessed across treatments by grouping individuals into categories ‘A’ (vigorous growth and optimal structure), ‘B’ (acceptable growth for field planting), ‘C’ (below the minimum quality threshold), and Dead (non-viable) (Fig. 1). Inoculation significantly altered the distribution of morphological categories, with the magnitude and nature of the response differing among genotypes.

For genotype Z, inoculation resulted in a pronounced shift in category distribution (chi-square test, *p* < 0.001). Inoculated seedlings showed a higher proportion of individuals classified as category A (69.9%) compared to the control (53.9%), accompanied by a marked reduction in category B (21.8% vs. 39.5%). Mortality remained low and did not differ significantly between treatments (7.2% in inoculated vs. 6.2% in control seedlings). Examination of standardized residuals indicated that categories A and B were the primary contributors to the overall chi-square signal.

A similarly strong response was observed for genotype Y (chi-square test, *p* < 0.001), for which inoculation was associated with both qualitative improvements in seedling vigor and reduced mortality. In this genotype, the proportion of dead seedlings decreased sharply from 21.8% in the control treatment to 6.7% following inoculation, while the combined proportion of seedlings in categories A and B increased accordingly. Standardized residuals identified categories A, B, and Dead as the main drivers of the observed differences.

In contrast, genotype X exhibited a more modest but still significant response to inoculation (chi-square test, *p* = 0.001). Inoculated seedlings showed a slight increase in category A (85.6% vs. 82.0% in the control) and a reduction in category B (5.2% vs. 8.4%), while mortality remained similar between treatments (8.6% in inoculated vs. 9.3% in control seedlings). Standardized residuals indicated that the significant effect for genotype X was primarily driven by changes in category B.

Overall, these results demonstrate that inoculation consistently influenced seedling morphological performance, although the intensity and specific response patterns were genotype dependent.

### Patterns in the endophytic bacterial composition of the root

**Fig. 2:**
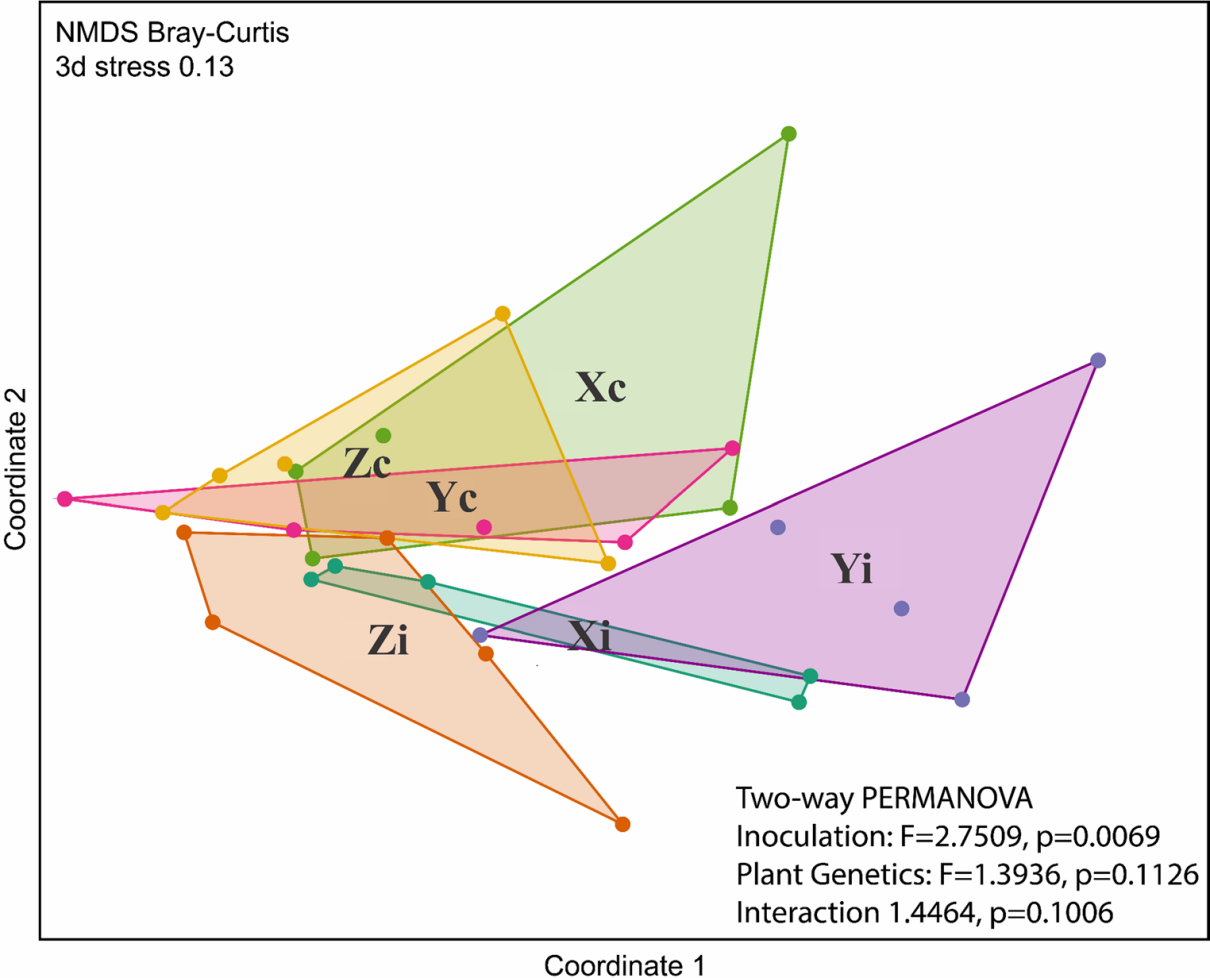
Non-metric Multidimensional Scaling (nMDS) with Bray–Curtis dissimilarity index of OTU distribution matrices in all samples. Treatments correspond to three *Eucalyptus* genotypes (X, Y, and Z), either inoculated (i) or non-inoculated control (c). Sample colors represent treatments as follows: Xc (light green), Xi (dark green), Yc (pink), Yi (purple), Zc (yellow), and Zi (orange). A two-way PERMANOVA was conducted for all samples, utilizing inoculation and genotype as factors

Non-metric multidimensional scaling (nMDS) based on Bray–Curtis dissimilarities revealed a significant shift in bacterial community structure in response to consortium inoculation (Two-way PERMANOVA, F = 2.7509, *p* = 0.0069) (Fig. 2). Inoculated treatments (Xi, Yi, and Zi) formed distinct clusters, separating from their respective controls (Xc, Yc, and Zc), which grouped more closely together. The F value indicates a moderate but consistent effect of inoculation on community composition. In contrast, plant genotype did not significantly influence bacterial assemblages (F = 1.3936, *p* = 0.1126), and no significant interaction between inoculation and genotype was detected (F = 1.4464, *p* = 0.1006).

The endophytic bacterial composition of *Eucalyptus* seedlings exhibited low richness and diversity across treatments. The average number of OTUs ranged from 180 to 200, while Shannon diversity values varied between 2.41 (Xi) and 2.67 (Yi), with no significant differences associated with plant genotype (Supplementary Fig. 1).

**Fig. 3:**
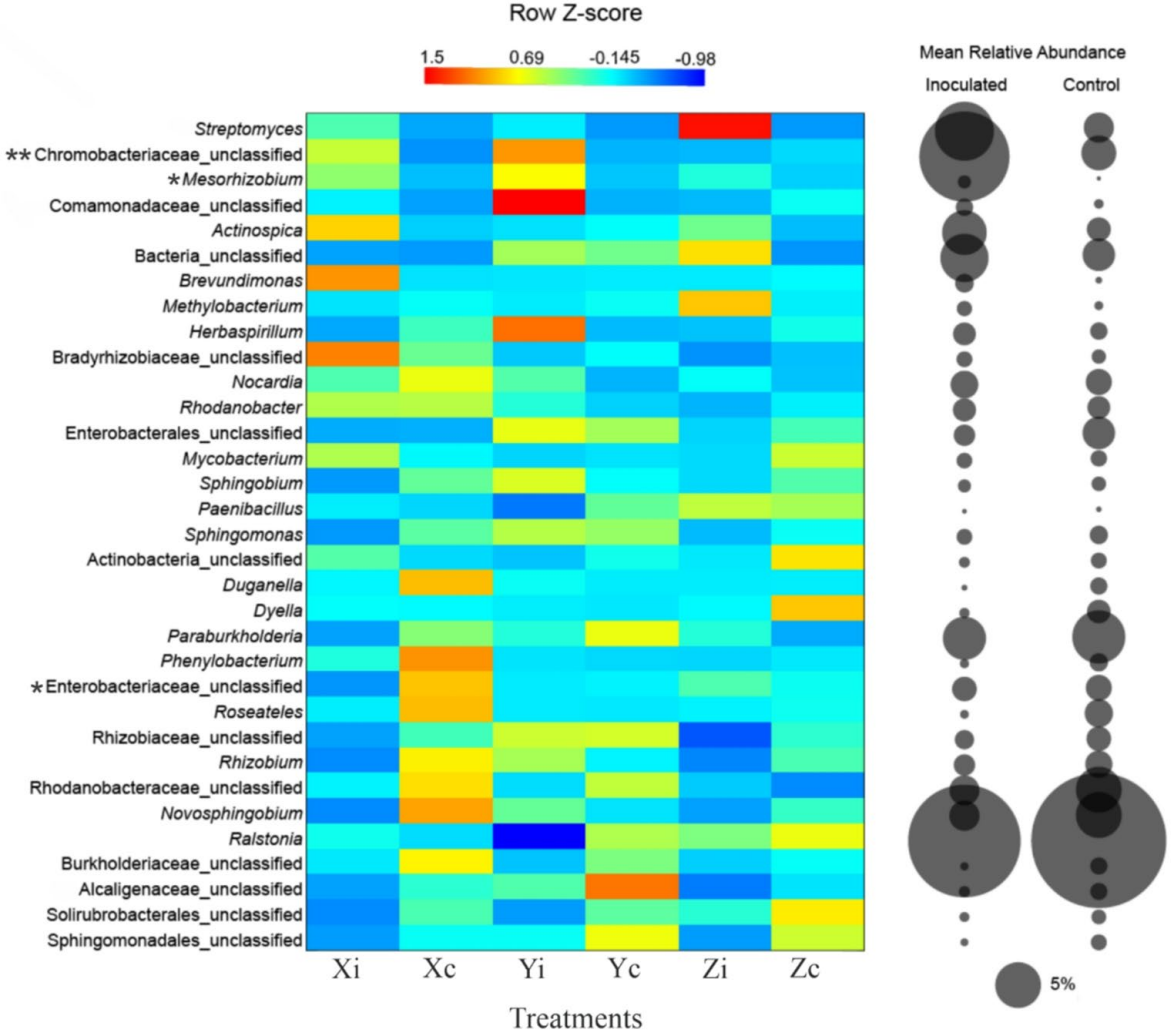
Heatmap showing relative differences in dominant bacterial genera detected in the root endophytic communities of *Eucalyptus* seedlings across treatments, calculated using row Z-scores (*n* = 5 per treatment). Black circles indicate the mean relative abundance of each genus (*n* = 15), with circle size proportional to abundance. Treatments correspond to three *Eucalyptus* genotypes (X, Y, and Z), either inoculated (i) or non-inoculated control (c). Exploratory differences among treatments were assessed using ANCOM-BC-2, considering the main effect of inoculation, genotype contrasts, and the inoculation × genotype interaction term. P-values were adjusted using the Benjamini–Hochberg false discovery rate (FDR), and all inferences are based on the resulting adjusted p-values (q-values). Asterisks (*) denote taxa showing exploratory differential abundance signals prior to sensitivity analysis, and double asterisks (**) indicate exploratory genotype-associated trends. No taxa were identified as robustly differentially abundant after ANCOM-BC-2 sensitivity analysis

At the phylum level, Proteobacteria and Actinobacteria dominated across all genotypes (Supplemental Fig. 2 and Supplemental Table 2). At lower taxonomic resolution, a total of 409 bacterial taxa were identified, predominantly at the genus level. The most abundant taxa (> 1% relative abundance) included *Ralstonia*, Chromobacteriaceae_unclassified, *Paraburkholderia*, *Streptomyces*, *Novosphingobium*, Rhodanobacteraceae_unclassified, *Actinospica*, Enterobacteriaceae_unclassified, and *Nocardia* (Fig. 3 and Supplemental Table 1).

Differential abundance analyses were evaluated using ANCOM-BC-2 on non-rarefied count data, applying a two-way model that included inoculation, genotype, and their interaction. No phyla were identified as robustly differentially abundant for the main effects of inoculation, genotype, or their interaction after false discovery rate (FDR) correction and ANCOM-BC-2 sensitivity analysis (diff_robust = FALSE for all taxa), indicating that shifts in community composition at the phylum level were not robustly supported under a compositionality-aware framework.

Similarly, no genera were detected as robustly differentially abundant for inoculation, genotype, or the inoculation × genotype interaction after sensitivity analysis. However, because the ANCOM-BC-2 robustness assessment is intentionally conservative, particularly for sparse count data with many zeros, we additionally examined taxa that were significant prior to sensitivity filtering as exploratory, non-robust trends.

Detailed results for taxa showing non-robust differential abundance signals under the main effect of inoculation are presented in Table S3. At the genus level, 11 taxa (including unclassified lineages) were associated with non-robust differential abundance patterns between inoculated and control seedlings. In the I vs. C contrast, Neisseriales_unclassified, *Mesorhizobium*,* Bosea*,* Rhodopseudomonas*,* Reyranella*, and Bacteroidota_unclassified showed positive log2FC values, indicating higher abundance in inoculated seedlings. In contrast, Enterobacteriaceae_unclassified, Pseudomonadaceae_unclassified, *Gryllotalpicola*, *Undibacter*, and *Thermoactinomyces* showed negative log2FC values, indicating higher abundance in control seedlings.

Regarding genotype effects in the global model, non-robust trends were detected for the Y vs. X contrast (13 genera), whereas no trends were observed for Z vs. X. A derived contrast from the global model further identified four genera (*Agrobacterium*,* Actinomadura*,* Actinoplanes*, and *Humibacter*) showing genotype-associated trends between Z and Y, with Agrobacterium exhibiting higher abundance in Z, while the remaining genera showed lower abundance in Z relative to Y. In addition, three genera (*Rhodopseudomonas*, *Reyranella*, and *Actinomadura*) exhibited non-robust trends for the inoculation × genotype interaction, suggesting genotype-dependent responses to inoculation. All non-robust signals are reported as exploratory and are provided in Supplementary Table 3. In genotypes Y and Z, inoculated seedlings tended to show lower relative abundance of the genus *Ralstonia* compared to control seedlings, with the most pronounced difference observed in genotype Y, where the mean relative abundance was approximately 34% lower than in the control treatment (Supplementary Fig. 3). However, this pattern was not identified as statistically significant in the compositionality-aware ANCOM-BC-2 analysis, and should therefore be interpreted as a descriptive, non-robust trend.

We specifically evaluated the relative abundance of the bacterial genera that composed the inoculated consortium. Overall, no consistent increase in the relative abundance of these genera was observed in the inoculated treatments, except for the genus *Mesorhizobium*, whose mean relative abundance was increased in the inoculated treatments when compared to the non-inoculated treatments (Fig. 3).

### Morphological responses of seedlings

**Fig. 4:**
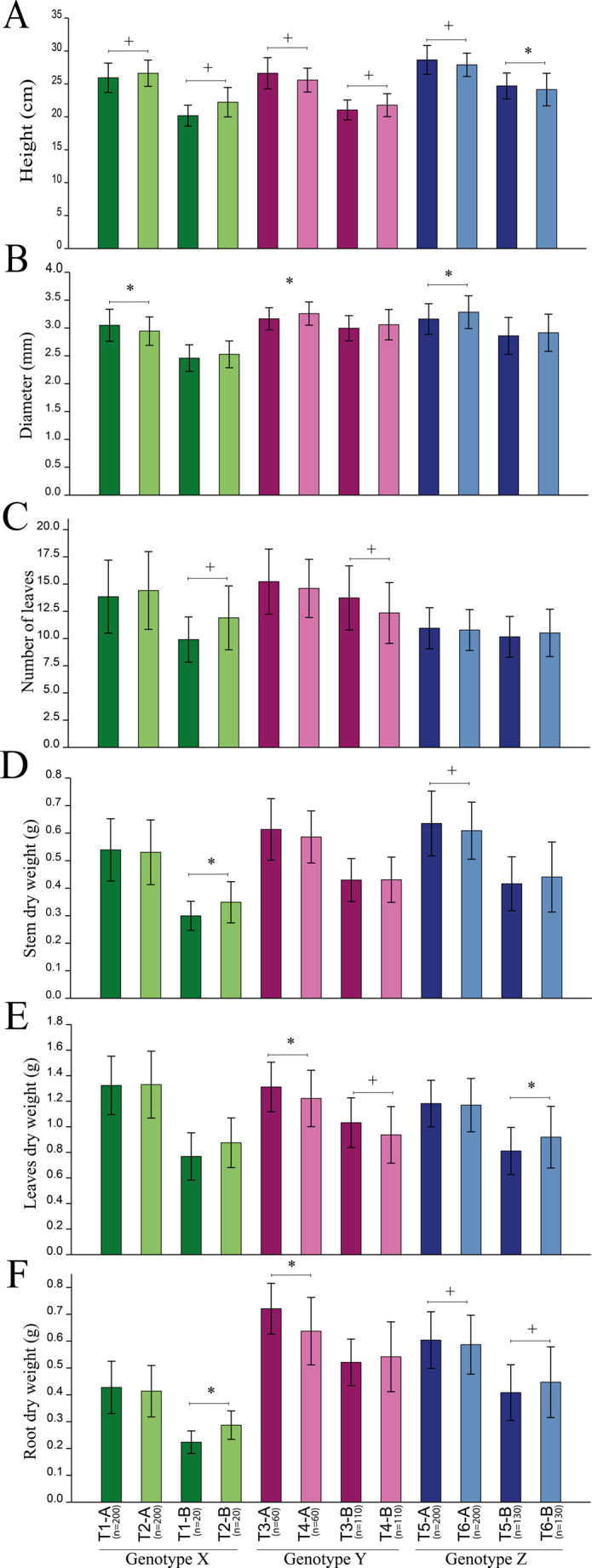
Morphological analyses of *Eucalyptus* seedlings subjected to different treatments. Graph ‘A’ represents height (cm), ‘B’ represents stem diameter (mm), ‘C’ represents the number of leaves, ‘D’ represents shoot dry weight, ‘E’ represents leaf dry weight, and graph ‘F’ represents root dry weight. Odd-numbered treatments correspond to inoculated seedlings, while even-numbered treatments are control groups. The final letter in each treatment code (A or B) represents seedling quality as determined by the company, where ‘A’ indicates excellent quality and ‘B’ indicates good quality. Normality was assessed using the Shapiro-Wilk test. Student’s *t*-test was applied to normally distributed data (marked with ‘*’), while the Mann-Whitney test was used for non-normal distributions (marked with ‘+’). Bars represent the mean ± standard deviation

We evaluated seedling development through morphological indicators, including plant height, stem diameter, number of leaves, and biomass allocation (shoot, leaf, and root dry weight). To capture potential interactions between inoculation and intrinsic seedling quality, plants were analyzed separately according to categories ‘A’ (excellent) and ‘B’ (good). Inoculation exerted a significant influence on both height and stem diameter across the three genotypes, though the direction of the effect depended on genotype and seedling quality class. In genotype X, category ‘A’ seedlings were taller in the control group, whereas inoculated seedlings displayed greater stem diameter. For genotype Y, category ‘A’ seedlings tended to be taller when inoculated, while category ‘B’ seedlings were taller in the control group (Fig. 4). Despite this variation in height, stem diameter was consistently larger in control seedlings of this genotype.

The number of leaves was significantly affected by inoculation only in genotypes X and Y, but in contrasting directions: control seedlings of genotype X exhibited more leaves, whereas inoculated seedlings of genotype Y showed superior leaf development (Fig. 4C). Regarding biomass, inoculation effects were trait- and genotype-specific. Stem dry weight was significantly higher only in genotype Z, restricted to category ‘A’ seedlings, where inoculated plants outperformed the controls. Leaf dry weight was enhanced by inoculation exclusively in genotype Y, in both categories ‘A’ and ‘B’ (Fig. 4D). Finally, root dry weight was significantly affected in genotypes Y and Z, but only among category ‘A’ seedlings, with inoculated plants presenting superior biomass accumulation (Fig. 4F).

Overall, these results demonstrate that inoculation differentially modulated morphological traits depending on genotype and seedling quality class, suggesting complex genotype × inoculation × quality interactions that affected growth allocation patterns (Fig. 4).

## Discussion

The use of bacterial inoculants represents a sustainable alternative to conventional fertilization, pest, and disease control methods, which could help to reduce chemical inputs, minimize environmental impacts, and enhance the long-term sustainability of agricultural systems. (Santos and Olivares 2021). In this study, we evaluated how the inoculation of a multi-strain bacterial consortium affects *Eucalyptus* seedling development and the composition of its root endophytic bacterial community. Overall, we demonstrate that the inoculation effect is genotype-dependent, capable of altering some microbiological features and able to improve seedling quality.

Although inoculation did not significantly modify bacterial richness or alpha diversity, it restructured the composition of root-associated bacterial communities, as indicated by nMDS ordination and PERMANOVA analyses. This pattern of community-level reorganization without concomitant changes in diversity indices is consistent with previous findings in microbial ecology, where functional rearrangement rather than taxonomic expansion drives plant–microbe interactions (Cardoso and Andreote 2016; Compant et al. 2019).

Despite these compositional shifts, the overall bacterial profiles remained similar among treatments, and no significant differences were detected among genotypes. Such uniformity may be explained by genetic proximity among the *Eucalyptus* clones, as well as by the homogeneous environmental and management conditions under which all seedlings were cultivated. Shared factors such as substrate composition, irrigation regime, and nursery microclimate are known to strongly constrain differentiation of bacterial composition (Zhang et al. 2022).

Among the taxa associated with inoculation, only *Mesorhizobium* exhibited a consistent increase across all inoculated treatments, suggesting successful root colonization and establishment in the endophytic environment of the seedlings. Considering the well-established role of *Mesorhizobium* spp. in biological nitrogen fixation, phytohormone production (e.g., IAA, cytokinins, gibberellins), and phosphate solubilization, its persistence within the endophytic compartment may provide long-term physiological. (Gopalakrishnan et al. 2015; García-Pérez et al. 2024; Camacho et al. 2025). Although *Mesorhizobium* increased in all inoculated treatments, the complete genome sequencing was not performed to track the strains after the experiment, which represents a limitation in confirming the persistence of the synthetic microbial community (SynCom) members throughout the entire experimental period. Future experiments incorporating this analysis would be important to confirm the presence of the SynCom member’s post-experiment. Even without confirming the presence of the inoculated members, their persistence within the endophytic compartment may provide long-term physiological benefits. The absence of a similar pattern for other inoculated strains aligns with the findings of Fazolato et al. (2025), who observed comparable outcomes in *Atriplex nummularia* inoculated with a bacterial consortium. In both cases, the inoculated strains appeared to exert their effects primarily through transient physiological modulation of plant metabolism, rather than through stable colonization or permanent restructuring of the native microbial community.

This phenomenon illustrates a recurrent feature of plant–microbe systems, where functional benefits precede taxonomic persistence, suggesting that growth promotion may result from short-term signaling or priming effects rather than long-term microbial establishment (Albright et al. 2022; Chen et al. 2022). The dissociation between microbial persistence and plant response indicates that inoculant efficacy depends less on niche occupation and more on the ability to trigger systemic plant responses, including hormonal modulation, antioxidant activation, and nutrient remobilization (Chakraborti et al. 2022; Kang et al. 2019). The bacterial strains used in this study were originally isolated as endophytes from *Eucalyptus* roots, which supported our decision to focus on the root endosphere as the target microhabitat. By investigating the same compartment from which the strains were obtained, we aimed to maximize ecological compatibility and the likelihood of successful colonization. Nevertheless, future studies exploring additional root-associated compartments, such as the rhizoplane, may provide complementary perspectives into plant–microbe interactions. Additionally, future research should incorporate metatranscriptomic and metabolomic analyses to elucidate the mechanistic underpinnings of these transient yet physiologically effective microbial influences on host plants.

It is also important to note that the bacterial composition was analyzed only at the end of the experiment, which means that the influence of the inoculant may have varied across different stages of plant development. Temporal fluctuations in bacterial richness and diversity often reflect dynamic responses to changes in management practices, nutrient availability, and the plant’s physiological stage (Li et al. 2021). Therefore, the absence of detectable increase in the average relative abundance of the consortium genera after 91 days does not necessarily indicate a lack of early colonization by the inoculated strains, nor does it preclude their transient influence on community structure and plant physiology (Moore et al. 2022). Future studies should investigate the early stages of microbial colonization, as initial community assembly may play a key role in determining long-term persistence and functional outcomes of inoculation.

The morphological outcomes observed in this study reinforce the need for genotype-specific approaches when using microbial consortia. In the case of genotype Y, the significant reduction in mortality (from 21.8% to 6.7%) led to an increase in the proportion of seedlings classified as ‘A’ and ‘B’, representing a direct benefit for production by reducing losses and increasing the availability of seedlings suitable for planting. For genotype Z, inoculation altered the quality distribution of seedlings, reducing the number of ‘B’ seedlings (from 39% to 21%) and increasing the proportion of ‘A’ seedlings (from 54% to 68%), suggesting a significant improvement in the final quality of this genotype. In contrast, genotype X exhibited minimal response to inoculation. It is worth noting that for the analysis, categories ‘A’ and ‘B’ were pooled together, as both represent seedlings that were deemed suitable for field planting. This decision reflects the practical application in forestry, where plants from both categories would be used for planting under normal conditions. Given this, and to provide a broader understanding of the bacterial community dynamics, we chose to pool these categories for amplicon sequencing, allowing us to focus on seedlings that are expected to perform similarly in field conditions. However, its baseline performance under control conditions was already high, leaving little physiological margin for additional improvement. This pattern underscores that the magnitude of microbial benefits depends on the host’s initial physiological status, and that genotypes with superior baseline performance might respond less markedly to inoculation. Such findings emphasize the importance of selecting responsive host genotypes when designing microbial consortia for forestry applications, ensuring that inoculation benefits are both measurable and agronomically relevant in a large field experiment conducted in a tree nursery.

Beyond changes in classification and survival, the effects of inoculation on growth varied between genotypes. Regarding height and diameter, the interaction between inoculation and classification indicated different responses: while in genotype X, control seedlings showed greater height, inoculated seedlings had a larger diameter. In genotype Y, inoculation favored the height of ‘A’ seedlings, while ‘B’ seedlings were taller in the control group, with consistently larger diameters also observed in control seedlings. These differences suggest that inoculation may influence the allocation of resources between height growth and stem thickening, depending on the genotype and experimental conditions. Despite the small observed differences, statistical analysis with large sample sizes confirmed their significance, suggesting reliable effects with potential biological relevance.

Stem dry weight was significantly higher only in ‘A’ seedlings of inoculated genotype Z, while leaf dry weight increased in genotype Y for both ‘A’ and ‘B’ seedlings. Additionally, root biomass was significantly higher in ‘A’ seedlings of genotypes Y and Z, suggesting that inoculation may promote root system development under certain conditions established by the company. Inoculation has been shown to consistently reprogram root and shoot architecture, as well as physiology and microbiomes (Khoso et al. 2024; Cunha et al. 2024). These results suggest that the effects of inoculation go far beyond simply promoting growth. Instead, inoculation influences plant development in a more complex way, impacting seedling architecture and its ability to adapt to field conditions (Raimam et al. 2024). This highlights the potential of inoculants to modify not only plant growth dynamics but also their structural and functional responses to environmental challenges (Rondina et al. 2020; Fonseca et al. 2022).

The moderate, yet consistent restructuring of the root bacterial composition following inoculation, without a loss of native diversity, suggests ecological compatibility between the introduced strains and the endogenous microbial community. This is an essential feature for long-term application of bioinoculants in forestry, as it ensures that microbiome stability and functional redundancy are preserved (Sessitsch et al. 2019). However, competition with the native microbiome may have prevented full establishment of the inoculant, an aspect that could be further investigated in future studies.

The observed reduction in average relative abundance of *Ralstonia*, when compared to non-inoculated treatments, supports the hypothesis that microbial consortia can serve as biocontrol agents through niche preemption, antibiosis, or induction of systemic resistance. These interactions could reduce dependency on chemical inputs and enhance soil health and phytosanitary resilience, aligning with the principles of sustainable silviculture (Eggers et al. 2020). The consistent higher average relative abundance of *Mesorhizobium*, a genus that includes diazotrophic lineages and has been previously reported as *nif*H-positive in association with *Eucalyptus* roots, supports the plausibility that inoculation may favor microbial pathways related to biological nitrogen inputs (Da Silva et al. 2014; Fonseca et al. 2018). Although direct nitrogen fixation was not measured in this study, such community-level signals are consistent with a potential contribution to nitrogen cycling, which, if confirmed by functional assays, could complement nutrient management strategies and contribute to reduced reliance on synthetic nitrogen fertilization.

Taken together, these findings indicate that the effects of bacterial inoculation operate at multiple levels, from shifts in community structure to changes in plant performance, with outcomes strongly dependent on host genotype. While the hypothesis that inoculation alters microbial community composition was partially supported, given that structural shifts were detected despite the absence of robust taxon-level changes, the hypothesis that the inoculated consortium would increase the relative abundance of its constituent genera was only weakly supported, with consistent enrichment observed only for *Mesorhizobium*. In contrast, the hypothesis that inoculation improves seedling development was supported, although dependent on host genotype.

## Conclusion

The findings of this study highlight the potential of bacterial consortia for plant growth promotion in forestry biotechnology. By simultaneously influencing bacterial community composition, and improving seedling quality, microbial inoculants represent a viable route toward low-input, high-resilience plantation systems. Nonetheless, the variability among genotypes emphasizes that inoculant development must consider host-specific compatibility, microbial persistence, and functional expression. Future research should integrate metagenomic and transcriptomic approaches to elucidate the functional pathways activated during plant–microbe interactions and to identify key microbial genes linked to growth promotion and stress mitigation. Long-term field trials are also necessary to assess the persistence of inoculated strains, their ecological safety, and their impact on soil functioning under variable climatic and edaphic conditions.

In summary, multi-strain bacterial inoculation offers a promising ecological and biotechnological tool to enhance *Eucalyptus* seedling performance, reduce mortality, and foster sustainable forestry practices. The differential responses observed here underline the need for tailored inoculant formulations that account for host genetics, environmental context, and microbial compatibility.

## Supplementary Information

Below is the link to the electronic supplementary material.

Supplementary Material 1 (DOCX 647 KB)

## Data Availability

Raw sequencing data was published in the NCBI Sequence Read Archive (SRA) under the Bioproject PRJNA1356519.

## Declarations

### Permission to reproduce material from other sources

The publisher or its licensor holds exclusive rights to this article under a publishing agreement with the author(s). Self-archiving of the accepted manuscript is allowed only as specified in that agreement and by applicable law.
