# Supplementary material for: Enhancing Eucalyptus seedling quality through multi-strain bacterial inoculation
Source: World J Microbiol Biotechnol. 2026 Apr 29;42(5):245. doi: 10.1007/s11274-026-04960-8 (PMC13124775; doi:10.1007/s11274-026-04960-8)
Supplement: Supplementary file 1 — Supplementary Material 1 (DOCX 647 KB) [file 11274_2026_4960_MOESM1_ESM.docx]

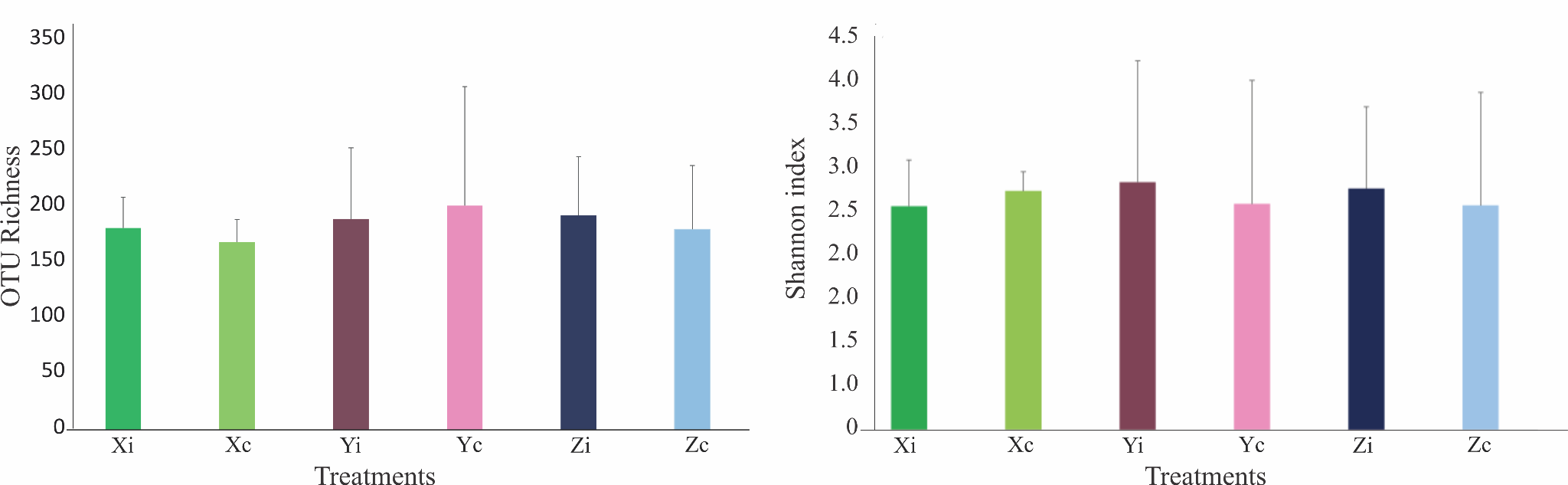


Supplementary Figure 1. Species richness (α-diversity) (A) and Shannon index (B) of samples derived from the roots of *Eucalyptus* seedlings from Treatment Xi, Treatment Xc, Treatment Xi, Treatment Yc, Treatment Zi and Treatment Zc.


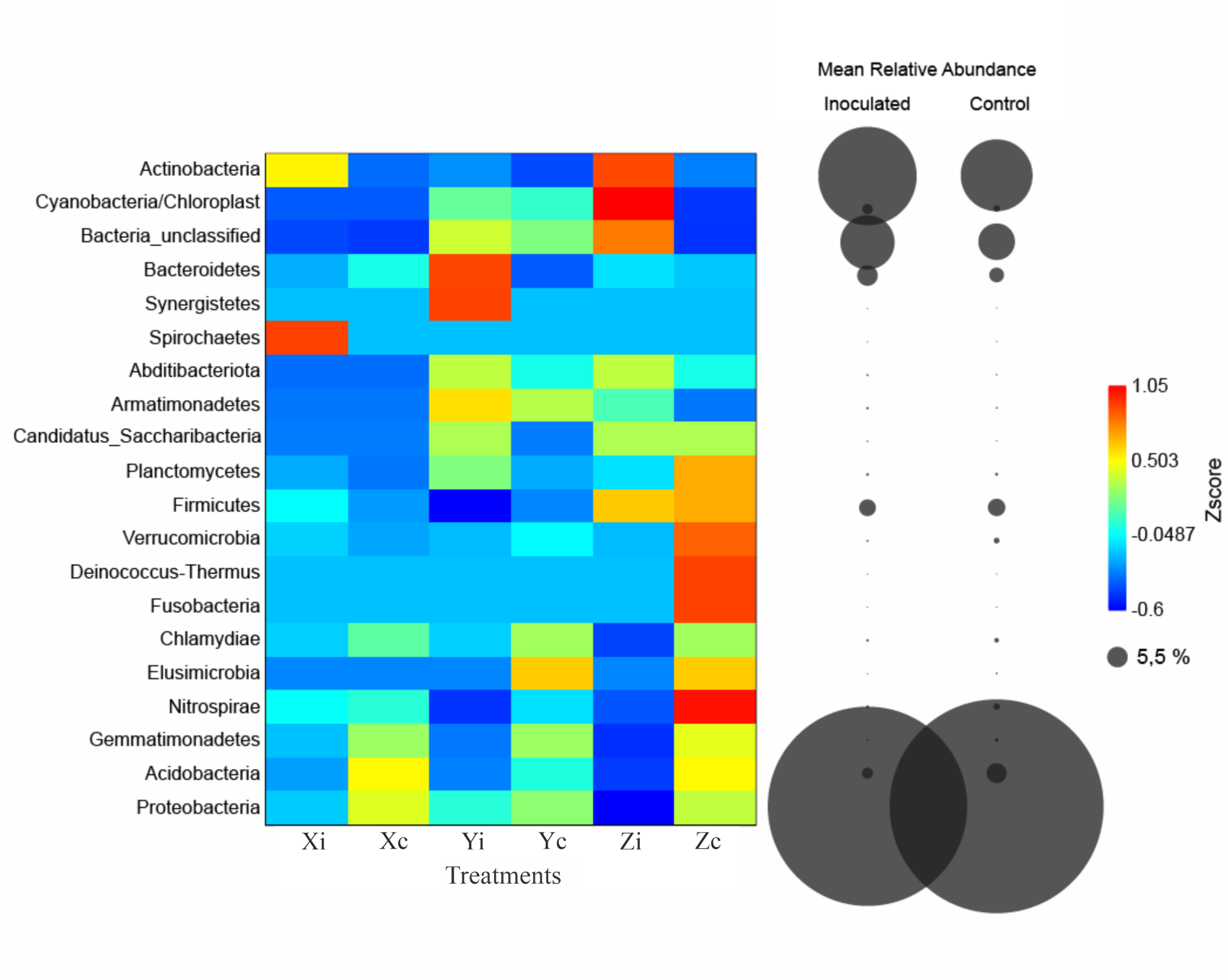


Supplementary Figure 2. Heatmap representing the average relative abundance of the main phyla within the Bacteria domain found in the roots of seedlings from Treatment Xi, Treatment Xc, Treatment Xi, Treatment Yc, Treatment Zi and Treatment Zc. The presence of an asterisk (*) indicates statistically significant differences in the treatment under the inoculation factor, while the presence of two asterisks (**) represents statistically significant differences in the genetic factor.


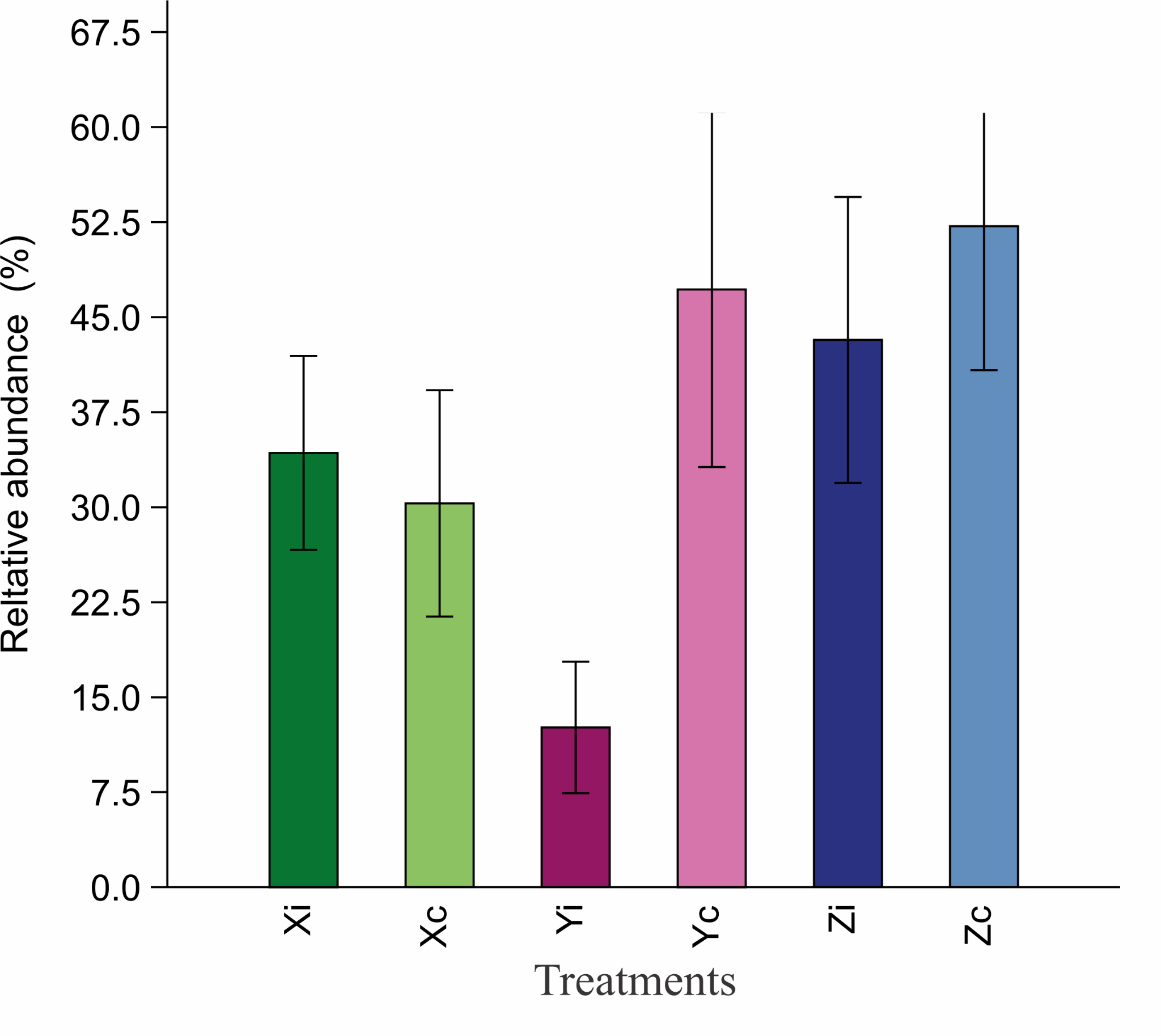


Supplementary Figure 3. Average relative abundance of the genus *Ralstonia* in Treatment Xi, Treatment Xc, Treatment Xi, Treatment Yc, Treatment Zi and Treatment Zc (n=5).


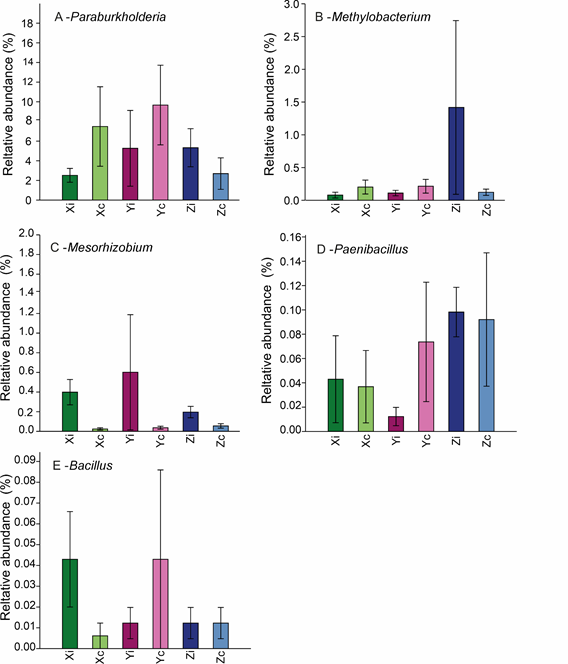


Supplemental Figure 4. Average relative abundance of all consortium components in Treatment Xi in dark green, Treatment 2 Xc in light green, Treatment Yi in purple, Treatment Yc in pink, Treatment Zi in dark blue, and Treatment 6 Zc in light blue. The consortium consists of five strains from the genera *Paraburkholderia* (A), *Methylobacterium* (B), *Mesorhizobium* (C*), Paenibacillus* (D), and *Bacillus* (E). The error bars represent standard deviation (n=5).

Supplemental table 1. Mean relative abundance (%) of dominant bacterial genera across treatments from Treatment Xi, Treatment Xc, Treatment Xi, Treatment Yc, Treatment Zi and Treatment Zc.

| **Bacterial genus** | **Treatments** | | | | | |
| --- | --- | --- | --- | --- | --- | --- |
|  | **Xi (%)** | **Xc (%)** | **Yi (%)** | **Yc (%)** | **Zi (%)** | **Zc (%)** |
| *Streptomyces* | 5.873 | 2.344 | 3.872 | 2.031 | 14.87 | 2.025 |
| Chromobacteriaceae_unclassified | 21.706 | 0.466 | 32.826 | 2.743 | 3.081 | 5.339 |
| *Mesorhizobium* | 0.399 | 0.025 | 0.601 | 0.037 | 0.196 | 0.055 |
| Comamonadaceae_unclassified | 0.313 | 0.11 | 1.602 | 0.153 | 0.172 | 0.368 |
| *Actinospica* | 8.168 | 1.117 | 1.516 | 2.123 | 4.357 | 0.755 |
| Bacteria_unclassified | 1.27 | 1.092 | 6.382 | 5.345 | 8.782 | 0.994 |
| *Brevundimonas* | 2.295 | 0.037 | 0.055 | 0.086 | 0.08 | 0.178 |
| *Methylobacterium* | 0.08 | 0.203 | 0.11 | 0.215 | 1.418 | 0.123 |
| *Herbaspirillum* | 0.123 | 1.074 | 3.234 | 0.239 | 0.288 | 0.792 |
| Bradyrhizobiaceae_unclassified | 1.154 | 0.644 | 0.344 | 0.448 | 0.246 | 0.331 |
| *Nocardia* | 2.025 | 3.664 | 2.087 | 0.473 | 1.289 | 0.626 |
| *Rhodanobacter* | 2.369 | 2.418 | 1.166 | 0.466 | 0.209 | 0.724 |
| Enterobacterales_unclassified | 0.209 | 0.239 | 3.424 | 2.737 | 0.614 | 1.755 |
| *Mycobacterium* | 0.878 | 0.485 | 0.405 | 0.436 | 0.417 | 0.939 |
| *Sphingobium* | 0.08 | 0.577 | 0.871 | 0.338 | 0.239 | 0.534 |
| *Paenibacillus* | 0.043 | 0.037 | 0.012 | 0.074 | 0.098 | 0.092 |
| *Sphingomonas* | 0.301 | 0.767 | 0.988 | 0.914 | 0.387 | 0.571 |
| Actinobacteria_unclassified | 0.571 | 0.135 | 0.055 | 0.331 | 0.19 | 1.276 |
| *Duganella* | 0.061 | 2.05 | 0.166 | 0.0 | 0.0 | 0.006 |
| *Dyella* | 0.331 | 0.264 | 0.123 | 0.061 | 0.258 | 3.553 |
| *Paraburkholderia* | 2.516 | 7.487 | 5.265 | 9.672 | 5.327 | 2.694 |
| *Phenylobacterium* | 0.411 | 2.117 | 0.117 | 0.055 | 0.043 | 0.141 |
| Enterobacteriaceae_unclassified | 0.018 | 4.94 | 1.025 | 1.117 | 2.16 | 1.399 |
| *Roseateles* | 0.166 | 4.946 | 0.123 | 0.086 | 0.233 | 0.583 |
| Rhizobiales_unclassified | 0.104 | 0.196 | 0.227 | 0.153 | 0.172 | 0.693 |
| *Rhizobium* | 0.601 | 2.51 | 2.007 | 1.123 | 0.577 | 1.54 |
| Rhodanobacteraceae_unclassified | 2.492 | 7.941 | 2.08 | 6.247 | 1.737 | 0.571 |
| *Novosphingobium* | 0.767 | 8.727 | 4.517 | 2.295 | 1.135 | 3.694 |
| Burkholderiaceae_unclassified | 0.215 | 1.068 | 0.104 | 0.651 | 0.135 | 0.295 |
| *Ralstonia* | 34.28 | 30.298 | 12.605 | 47.186 | 43.197 | 52.169 |
| Alcaligenaceae_unclassified | 0.184 | 0.473 | 0.552 | 1.209 | 0.104 | 0.325 |
| Solirubrobacterales_unclassified | 0.147 | 0.405 | 0.172 | 0.43 | 0.356 | 0.669 |
| Sphingomonadales_unclassified | 0.068 | 0.288 | 0.288 | 0.773 | 0.068 | 0.7 |

Supplemental table 2. Mean relative abundance (%) of dominant bacterial phyla across treatments from Treatment Xi, Treatment Xc, Treatment Xi, Treatment Yc, Treatment Zi and Treatment Zc.

| **Bacterial phylum** | **Treatments** | | | | | |
| --- | --- | --- | --- | --- | --- | --- |
|  | **Xi (%)** | **Xc (%)** | **Yi (%)** | **Yc (%)** | **Zi (%)** | **Zc (%)** |
| Actinobacteria | 19.755 | 9.745 | 10.641 | 8.917 | 23.909 | 10.218 |
| Cyanobacteria/Chloroplast | 0.043 | 0.043 | 0.172 | 0.147 | 0.368 | 0.025 |
| Bacteria_unclassified | 1.27 | 1.092 | 6.382 | 5.345 | 8.782 | 0.994 |
| Bacteroidetes | 0.356 | 0.583 | 1.491 | 0.172 | 0.466 | 0.411 |
| Synergistetes | 0 | 0 | 0.006 | 0 | 0 | 0 |
| Spirochaetes | 0.006 | 0 | 0 | 0 | 0 | 0 |
| Abditibacteriota | 0 | 0 | 0.012 | 0.006 | 0.012 | 0.006 |
| Armatimonadetes | 0 | 0 | 0.025 | 0.018 | 0.012 | 0 |
| Candidatus Saccharibacteria | 0 | 0 | 0.006 | 0 | 0.006 | 0.006 |
| Planctomycetes | 0.006 | 0 | 0.031 | 0.006 | 0.012 | 0.055 |
| Firmicutes | 0.503 | 0.381 | 0.141 | 0.356 | 0.878 | 0.914 |
| Verrucomicrobia | 0.012 | 0 | 0.006 | 0.025 | 0.006 | 0.141 |
| Deinococcus–Thermus | 0 | 0 | 0 | 0 | 0 | 0.006 |
| Fusobacteria | 0 | 0 | 0 | 0 | 0 | 0.006 |
| Chlamydiae | 0.018 | 0.031 | 0.018 | 0.037 | 0.006 | 0.037 |
| Elusimicrobia | 0 | 0 | 0 | 0.012 | 0 | 0.012 |
| Nitrospirae | 0.043 | 0.049 | 0.006 | 0.037 | 0.012 | 0.129 |
| Gemmatimonadetes | 0.012 | 0.031 | 0.006 | 0.031 | 0 | 0.037 |
| Acidobacteria | 0.282 | 0.853 | 0.233 | 0.485 | 0.123 | 0.853 |
| Proteobacteria | 77.693 | 87.192 | 80.822 | 84.406 | 65.407 | 86.149 |

Supplementary Table 3. Exploratory (non-robust) differential abundance signals detected by ANCOM-BC-2 at the Genus-level taxa (including unclassified lineages). The table lists taxa with significant differential abundance prior to ANCOM-BC-2 sensitivity filtering (diff = TRUE) that did not pass the robustness criterion (diff_robust = FALSE). Results are shown for the main effect of inoculation (I vs C), genotype contrasts (Y vs X), the derived genotype contrast (Z vs Y), and the inoculation × genotype interaction term. Reported effect sizes correspond to ANCOM-BC-2 log-fold changes, and p-values were adjusted using the Benjamini–Hochberg FDR procedure. These results should be interpreted as exploratory trends.

| **Taxon** | **Effect** | **Contrast** | **log2FC** | **q_value** | **Robust** |
| --- | --- | --- | --- | --- | --- |
| Enterobacteriaceae_unclassified | Inoculation | I vs C | -3.14 | 0.027 | FALSE |
| Neisseriales_unclassified | Inoculation | I vs C | 3.65 | 0.002 | FALSE |
| *Mesorhizobium* | Inoculation | I vs C | 2.77 | 0.019 | FALSE |
| *Bosea* | Inoculation | I vs C | 2.40 | 0.033 | FALSE |
| Pseudomonadaceae_unclassified | Inoculation | I vs C | -1.65 | 0.039 | FALSE |
| *Rhodopseudomonas* | Inoculation | I vs C | 2.52 | 0.033 | FALSE |
| *Gryllotalpicola* | Inoculation | I vs C | -1.96 | 0.033 | FALSE |
| *Reyranella* | Inoculation | I vs C | 2.54 | 0.037 | FALSE |
| Bacteroidota_unclassified | Inoculation | I vs C | 2.06 | 0.033 | FALSE |
| *Undibacter* | Inoculation | I vs C | -4.08 | 0.008 | FALSE |
| *Thermoactinomyces* | Inoculation | I vs C | -1.83 | 0.044 | FALSE |
| Chromobacteriaceae_unclassified | Genotype | Y vs X | 1.54 | 0.029 | FALSE |
| Pseudomonadota_unclassified | Genotype | Y vs X | -0.99 | 0.040 | FALSE |
| *Agrobacterium* | Genotype | Y vs X | -0.93 | 0.001 | FALSE |
| *Staphylococcus* | Genotype | Y vs X | 0.88 | 0.0415 | FALSE |
| *Chromobacterium* | Genotype | Y vs X | 1.41 | 0.0009 | FALSE |
| *Reyranella* | Genotype | Y vs X | 2.32 | 0.0004 | FALSE |
| Bacteroidota_unclassified | Genotype | Y vs X | 1.04 | 0.0008 | FALSE |
| *Alicyclobacillus* | Genotype | Y vs X | -0.66 | 0.007 | FALSE |
| Erwiniaceae_unclassified | Genotype | Y vs X | 2.00 | 0.009 | FALSE |
| *Actinomadura* | Genotype | Y vs X | 3.61 | 0.0001 | FALSE |
| *Undibacter* | Genotype | Y vs X | -2.83 | 6.1147e-05 | FALSE |
| Nocardioidaceae_unclassified | Genotype | Y vs X | 1.24 | 0.005 | FALSE |
| *Humibacter* | Genotype | Y vs X | 1.48 | 0.0009 | FALSE |
| *Agrobacterium* | Genotype | Z vs Y (derived) | 1.80 | 0.0371 | FALSE |
| *Actinomadura* | Genotype | Z vs Y (derived) | -3.14 | 0.001 | FALSE |
| *Actinoplanes* | Genotype | Z vs Y (derived) | -4.06 | 0.0002 | FALSE |
| *Humibacter* | Genotype | Z vs Y (derived) | -2.19 | 0.006 | FALSE |
| *Rhodopseudomonas* | Interaction | Inoculation √ó Genotype (Y term) | -4.39 | 0.034 | FALSE |
| *Reyranella* | Interaction | Inoculation √ó Genotype (Y term) | -4.74 | 0.030 | FALSE |
| *Actinomadura* | Interaction | Inoculation √ó Genotype (Y term) | -5.07 | 0.030 | FALSE |
